# Supplementary material for: Allosteric regulation and crystallographic fragment screening of SARS-CoV-2 NSP15 endoribonuclease
Source: Nucleic Acids Res. 2023 Apr 28;51(10):5255–70. doi: 10.1093/nar/gkad314 (PMC10250223; doi:10.1093/nar/gkad314)
Supplement: gkad314_Supplemental_Files [file gkad314_supplemental_files.zip › Supplementary datasets.zip!/81/Refine_9/01-BUSTER/Cycle-3/shell.01/R_Rfree.html]

# Rxpct factors in resolution bins at cycle 0


|  |  |  |  |  |  |  |  |  |
| --- | --- | --- | --- | --- | --- | --- | --- | --- |
|  | **<d> (Å)**  Rxpct\_Work | Rxpct\_Free | Rxpct\_All | |  |  |  |  |
|  | 15.0454 | 0.2866 | 0.2273 | 0.2844 |  |
|  | 8.6865 | 0.2138 | 0.2289 | 0.2147 |  |
|  | 6.7285 | 0.2652 | 0.2450 | 0.2641 |  |
|  | 5.6866 | 0.2958 | 0.2909 | 0.2957 |  |
|  | 5.0151 | 0.2400 | 0.2619 | 0.2414 |  |
|  | 4.5364 | 0.2292 | 0.2583 | 0.2306 |  |
|  | 4.1728 | 0.2423 | 0.2143 | 0.2408 |  |
|  | 3.8847 | 0.2661 | 0.2511 | 0.2654 |  |
|  | 3.6491 | 0.2732 | 0.2478 | 0.2719 |  |
|  | 3.4517 | 0.2783 | 0.2627 | 0.2775 |  |
|  | 3.2832 | 0.2805 | 0.2980 | 0.2816 |  |
|  | 3.1372 | 0.2715 | 0.2714 | 0.2715 |  |
|  | 3.0091 | 0.2599 | 0.2710 | 0.2603 |  |
|  | 2.8955 | 0.2450 | 0.2467 | 0.2450 |  |
|  | 2.7939 | 0.2296 | 0.2116 | 0.2288 |  |
|  | 2.7022 | 0.2105 | 0.2107 | 0.2105 |  |
|  | 2.6191 | 0.1904 | 0.2062 | 0.1913 |  |
|  | 2.5431 | 0.1816 | 0.1824 | 0.1817 |  |
|  | 2.4735 | 0.1699 | 0.1631 | 0.1695 |  |
|  | 2.4092 | 0.1755 | 0.1605 | 0.1748 |  |
|  | **Overall** | 0.2497 | 0.2447 | 0.2494 |  |

# Rxpct factors in resolution bins at cycle 1


|  |  |  |  |  |  |  |  |  |
| --- | --- | --- | --- | --- | --- | --- | --- | --- |
|  | **<d> (Å)**  Rxpct\_Work | Rxpct\_Free | Rxpct\_All | |  |  |  |  |
|  | 15.0454 | 0.2864 | 0.2267 | 0.2842 |  |
|  | 8.6865 | 0.2152 | 0.2313 | 0.2161 |  |
|  | 6.7285 | 0.2691 | 0.2489 | 0.2679 |  |
|  | 5.6866 | 0.2974 | 0.2952 | 0.2974 |  |
|  | 5.0151 | 0.2399 | 0.2620 | 0.2413 |  |
|  | 4.5364 | 0.2264 | 0.2593 | 0.2281 |  |
|  | 4.1728 | 0.2405 | 0.2157 | 0.2392 |  |
|  | 3.8847 | 0.2632 | 0.2487 | 0.2625 |  |
|  | 3.6491 | 0.2709 | 0.2488 | 0.2698 |  |
|  | 3.4517 | 0.2766 | 0.2608 | 0.2758 |  |
|  | 3.2832 | 0.2787 | 0.2989 | 0.2800 |  |
|  | 3.1372 | 0.2698 | 0.2667 | 0.2697 |  |
|  | 3.0091 | 0.2583 | 0.2700 | 0.2588 |  |
|  | 2.8955 | 0.2410 | 0.2433 | 0.2411 |  |
|  | 2.7939 | 0.2278 | 0.2151 | 0.2272 |  |
|  | 2.7022 | 0.2091 | 0.2064 | 0.2090 |  |
|  | 2.6191 | 0.1912 | 0.2061 | 0.1921 |  |
|  | 2.5431 | 0.1815 | 0.1837 | 0.1816 |  |
|  | 2.4735 | 0.1723 | 0.1632 | 0.1718 |  |
|  | 2.4092 | 0.1790 | 0.1678 | 0.1785 |  |
|  | **Overall** | 0.2489 | 0.2451 | 0.2487 |  |

# Rxpct factors in resolution bins at cycle 2


|  |  |  |  |  |  |  |  |  |
| --- | --- | --- | --- | --- | --- | --- | --- | --- |
|  | **<d> (Å)**  Rxpct\_Work | Rxpct\_Free | Rxpct\_All | |  |  |  |  |
|  | 15.0454 | 0.2846 | 0.2257 | 0.2824 |  |
|  | 8.6865 | 0.2136 | 0.2314 | 0.2146 |  |
|  | 6.7285 | 0.2630 | 0.2436 | 0.2619 |  |
|  | 5.6866 | 0.2895 | 0.2893 | 0.2894 |  |
|  | 5.0151 | 0.2354 | 0.2581 | 0.2369 |  |
|  | 4.5364 | 0.2260 | 0.2618 | 0.2278 |  |
|  | 4.1728 | 0.2404 | 0.2174 | 0.2391 |  |
|  | 3.8847 | 0.2636 | 0.2496 | 0.2630 |  |
|  | 3.6491 | 0.2716 | 0.2490 | 0.2705 |  |
|  | 3.4517 | 0.2769 | 0.2639 | 0.2762 |  |
|  | 3.2832 | 0.2779 | 0.3006 | 0.2793 |  |
|  | 3.1372 | 0.2698 | 0.2674 | 0.2697 |  |
|  | 3.0091 | 0.2583 | 0.2703 | 0.2588 |  |
|  | 2.8955 | 0.2448 | 0.2438 | 0.2448 |  |
|  | 2.7939 | 0.2296 | 0.2141 | 0.2289 |  |
|  | 2.7022 | 0.2094 | 0.2081 | 0.2093 |  |
|  | 2.6191 | 0.1912 | 0.2109 | 0.1924 |  |
|  | 2.5431 | 0.1853 | 0.1889 | 0.1855 |  |
|  | 2.4735 | 0.1717 | 0.1657 | 0.1714 |  |
|  | 2.4092 | 0.1793 | 0.1654 | 0.1787 |  |
|  | **Overall** | 0.2478 | 0.2452 | 0.2476 |  |

# Rxpct factors in resolution bins at cycle 3


|  |  |  |  |  |  |  |  |  |
| --- | --- | --- | --- | --- | --- | --- | --- | --- |
|  | **<d> (Å)**  Rxpct\_Work | Rxpct\_Free | Rxpct\_All | |  |  |  |  |
|  | 15.0454 | 0.2850 | 0.2240 | 0.2827 |  |
|  | 8.6865 | 0.2152 | 0.2345 | 0.2163 |  |
|  | 6.7285 | 0.2652 | 0.2409 | 0.2638 |  |
|  | 5.6866 | 0.2903 | 0.2934 | 0.2904 |  |
|  | 5.0151 | 0.2366 | 0.2585 | 0.2380 |  |
|  | 4.5364 | 0.2238 | 0.2608 | 0.2256 |  |
|  | 4.1728 | 0.2402 | 0.2155 | 0.2389 |  |
|  | 3.8847 | 0.2632 | 0.2460 | 0.2624 |  |
|  | 3.6491 | 0.2703 | 0.2467 | 0.2692 |  |
|  | 3.4517 | 0.2750 | 0.2640 | 0.2745 |  |
|  | 3.2832 | 0.2770 | 0.2987 | 0.2784 |  |
|  | 3.1372 | 0.2673 | 0.2656 | 0.2673 |  |
|  | 3.0091 | 0.2549 | 0.2647 | 0.2553 |  |
|  | 2.8955 | 0.2414 | 0.2448 | 0.2416 |  |
|  | 2.7939 | 0.2268 | 0.2120 | 0.2261 |  |
|  | 2.7022 | 0.2070 | 0.2039 | 0.2069 |  |
|  | 2.6191 | 0.1879 | 0.2062 | 0.1890 |  |
|  | 2.5431 | 0.1808 | 0.1831 | 0.1810 |  |
|  | 2.4735 | 0.1709 | 0.1641 | 0.1706 |  |
|  | 2.4092 | 0.1772 | 0.1680 | 0.1768 |  |
|  | **Overall** | 0.2469 | 0.2440 | 0.2468 |  |
